# Force and Velocity Analysis of Particles Manipulated by Toroidal Vortex on Optoelectrokinetic Microfluidic Platform

**DOI:** 10.3390/mi13122245

**Published:** 2022-12-17

**Authors:** Sheng-Jie Zhang, Zong-Rui Yang, Ju-Nan Kuo

**Affiliations:** Department of Automation Engineering, National Formosa University, No. 64, Wenhua Rd., Huwei, Yunlin 632, Taiwan

**Keywords:** velocity analysis, toroidal vortex, optoelectrokinetic, REP, drag force, trapping force

## Abstract

The rapid electrokinetic patterning (REP) technique has been demonstrated to enable dynamic particle manipulation in biomedical applications. Previous studies on REP have generally considered particles with a size less than 5 μm. In this study, a REP platform was used to manipulate polystyrene particles with a size of 3~11 μm in a microfluidic channel sandwiched between two ITO conductive glass plates. The effects of the synergy force produced by the REP electrothermal vortex on the particle motion were investigated numerically for fixed values of the laser power, AC driving voltage, and AC driving frequency, respectively. The simulation results showed that the particles were subject to a competition effect between the drag force produced by the toroidal vortex, which prompted the particles to recirculate in the bulk flow adjacent to the laser illumination spot on the lower electrode, and the trapping force produced by the particle and electrode interactions, which prompted the particles to aggregate in clusters on the surface of the illuminated spot. The experimental results showed that as the laser power increased, the toroidal flow range over which the particles circulated in the bulk flow increased, while the cluster range over which the particles were trapped on the electrode surface reduced. The results additionally showed that the particle velocity increased with an increasing laser power, particularly for particles with a smaller size. The excitation frequency at which the particles were trapped on the illuminated hot-spot reduced as the particle size increased. The force and velocity of polystyrene particles by the REP toroidal vortex has implications for further investigating the motion behavior at the biological cell level.

## 1. Introduction

Micro-manipulation technology is widely used in the biotechnology and bio-medical fields nowadays. Furthermore, many operations, such as trapping, transportation, concentration, and sorting of micro/nano-scaled particles, are now routinely conducted on microfluidic lab-on-a chip devices [1,2] and medical diagnostic platforms [3]. Microfluidics technology provides the ability to manipulate nanoliter volumes of sample fluid with an extremely high precision, and thus has many benefits for biochemical analysis, including a lower cost, a more straightforward operation, a greater sensitivity, and a higher throughput [4]. However, these advantages come at the expense of a greater complexity of the manipulation process compared to that required for traditional macroscale instruments. Therefore, effective techniques for manipulating large numbers of particles rapidly and accurately are essential to further advance the biomedical engineering field [5].

Various techniques for manipulating micro- and nano-sized particles or biological cells have been proposed, including dielectrophoresis [6], plasmonic traps [7], optical tweezers [8], acoustic [9], magnetic [10], and electrokinetics tweezers [11]. However, dielectrophoresis requires the use of fixed electrodes, which limits its application. Moreover, dielectrophoresis is restricted to the manipulation of large particles since the dielectrophoresis force is proportional to the cube of the particle radius [12]. Meanwhile, plasmonic traps require plasmonic nanostructures, which are expensive to fabricate and generate a trapping force over only a very limited distance (e.g., several nanometers from the nanostructure) [13]. Finally, optical tweezers and electrokinetics tweezers have poor resolution and throughput for particle manipulation [14].

Optoelectrokinetic techniques combine optical and electrokinetic effects to yield high resolution, high throughput, and programmable manipulation. The optical control of electrokinetic forces is traditionally performed using either optoelectronic tweezers (OET) or optically induced dielectrophoresis (ODEP) [15]. However, in recent years, rapid electrokinetic patterning (REP), in which particle manipulation is achieved by changing the electrical properties of the medium in which the particles are suspended [16], has emerged as a powerful noninvasive and programmable alternative for a wide variety of bioanalysis applications [17,18,19,20]. The REP technique uses a parallel plate electrode to generate a uniform alternating electric field and a highly focused laser beam to create localized hot-spots on the surface of the lower electrode. The localized hotspots induce the formation of electrothermal microvortex structures [21], which facilitate the rapid manipulation of the analyte at the corresponding location. The authors in [22] examined the toroidal vortex structure generated by circular hot-spots using wavefront deformation particle-tracking velocimetry and particle image velocimetry, respectively. The results showed that the toroidal vortex strength increased with temperature and applied electric potential. However, the effects of the laser power on the characteristics of the toroidal vortex structures were not considered.

The parallel plate electrodes used in REP platforms are generally fabricated from indium tin oxide (ITO) coated glass, which allows for the simultaneous application of both an AC electric field and highly localized infrared laser illumination [23]. ITO is strongly absorbent in the infrared range, and the resulting optical landscape produces high thermal gradients. When these gradients occur in the presence of an electric field, toroidal electrothermal flows are produced, which offer the potential to realize a wide variety of optoelectrokinetic applications [24]. The authors in [25,26] used the hydrodynamic drag force produced by the toroidal vortex to transport the particles toward the electrode surface, where they were subsequently trapped by the local electrode-particle interactions. The particles were then instantaneously dispersed by turning the laser off, thereby terminating the electrothermal flow. It was shown in a recent study that REP exerts a force on the order of several femtonewtons on micro- and nanoparticles during the manipulation process, and the transverse trapping force produced by REP originates from the axisymmetric Stokes drag force induced by the toroidal electrothermal vortex [27]. However, the exact roles played by the hydrodynamic drag force and trapping force in manipulating the particles between the electrodes are still unclear. Previous studies on REP have generally considered only small particles with a size of around 0.1~5 μm, and the bottom plate of the chip was fabricated by coating 150 nm gold as a conductive layer on a glass substrate [18]. The authors in [19] summarize a variety of differently structured optoelectrokinetic chips, followed by a discussion on how they are fabricated and the ways in which they work. Furthermore, while the particle velocity induced by the electrothermal microvortex structures in REP has been analyzed using both wavefront deformation particle-tracking velocimetry [21] and particle image velocimetry [28,29], these studies have focused only on the area of the laser illumination spot and the particles flowing toward this spot.

The present study conducts a numerical investigation regarding the effects of the electrothermal vortex induced in REP on the dynamic behavior of polystyrene particles with a size of 3~11 μm confined within a 100-μm microfluidic channel. The simulations focus specifically on the competing effects of the hydrodynamic drag force and trapping force on the particle motion under a constant setting of the laser power, AC voltage, and AC frequency, respectively. Experiments are then performed to examine the effects of the laser power and particle size on the toroidal flow range (i.e., the range over which the particles circulate within the bulk flow in the vicinity of the heated spot), cluster range (i.e., the range over which the particles are trapped in clusters on the electrode surface), and particle velocity. Further experiments are then conducted to investigate the excitation frequency required to produce a trapping effect for particles of different sizes. Finally, a voltage modulation technique is employed to perform the size-dependent sorting of the particles.

## 2. Theory

REP is a noninvasive technique involving the simultaneous application of a uniform AC electric field and an optical laser to produce a toroidal vortex with which to manipulate the particles. The optoelectrokinetic effect comprises four main flow components, namely AC electrothermal (ACET), AC electroosmosis (ACEO), dielectrophoresis (DEP), and thermophoresis. The details of each component are briefly described in the following sections.

### 2.1. AC Electrothermal

Electrothermal flow occurs as a result of heating the ITO surface by laser irradiation in the presence of an electric field. In particular, when a high-intensity infrared laser beam is projected onto the ITO surface, it heats the illuminated area, which in turn heats the surrounding bulk fluid. Consequently, gradients of conductivity and permittivity are produced, which prompt the movement of the ions in the liquid medium. When coupled with an applied AC electric field, an electrothermal flow is induced with the following time-averaged form [30]:

(1)         FE=12Re[σε(α−β)σ+iωε(∇T·E)E*−12εα|E|2∇T]

where Re is the real part of [], **E** is the electric field, and E* is the complex conjugate. In addition, *ω* is the frequency of the AC signal, *ε* is the permittivity of the fluid, and *α* and *β* are (1/*ε*)(∂*ε*/∂*T*) and (1/*σ*)(∂*σ*/∂*T*), respectively [31]. The first term in Equation (1) is the Coulomb force, while the second term is the dielectric force. Equation (1) clearly shows that the formation of localized temperature gradients leads to electrical forcing.

### 2.2. AC Electroosmosis

Electrode surfaces generally have a net charge. When the electrode structures are subjected to an AC voltage, a flow of the aqueous solution is induced at the solid liquid interface due to the effect of the AC field on the charges induced by itself at the electrode and electrolyte interface (i.e., the charges induced in the electrical double layer). This phenomenon is known as AC electroosmosis (ACEO) and is described by the following time-averaged expression [32]:

(2) 〈v〉=12Re[σqEt*λDη]

where *σ_q_* is the charge density in the double layer, *λ_D_* is the Debye length, $E_{t}^{*}$ is the complex conjugate of the tangential electric field, and *η* is the viscosity of the medium.

### 2.3. Dielectrophoresis

When a polarizable particle is suspended in a non-uniform electric field, the electric field polarizes the particle, and the poles then experience a force acting along the field lines, which can be either attractive or repulsive depending on the orientation of the dipole. Since the field is non-uniform, the pole experiencing the greatest electric field dominates the other, and the particle starts to move. For a homogeneous dielectric spherical particle immersed in a conductive medium, the time-averaged DEP force can be expressed as [33]

(3) $$F_{DEP}=2\pi a^{3}\varepsilon_{m}Re\left[CM\right]\nabla E^{2}$$

where *a* is the radius of the particle, *ε_m_* is the permittivity of the medium, *Re*[*CM*] is the real part of the complex Clausius–Mossotti factor, and *E* is the amplitude of the electric field.

### 2.4. Thermophoresis

Thermophoresis refers to the movement of suspended particles through a fluid under the effect of an applied thermal gradient. In particular, the thermal gradient causes the particle to experience a net force in the direction of decreasing temperature (i.e., positive thermophoresis) since the molecules impacting the particle on opposite sides through thermal motion have different average velocities as a result of their different temperatures. The effective thermophoresis force can be written as follows [34,35]:

(4) $$F_{T}=\gamma v_{T}=-k_{B}TS_{T}\nabla T$$

where *k_B_* is the Boltzmann constant, *T* is the temperature, and the ratio *S_T_* = *D_T_*/*D*, also known as the Soret coefficient, quantifies the strength and direction of the colloidal thermophoresis. Note that *D_T_* and *D* are the thermal diffusion coefficient and Fickian diffusion coefficient, respectively.

## 3. Materials and Methods

### 3.1. Chip Fabrication

**Figure 1:**
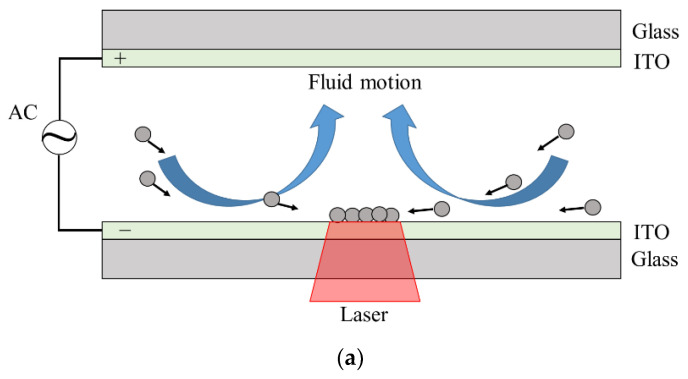

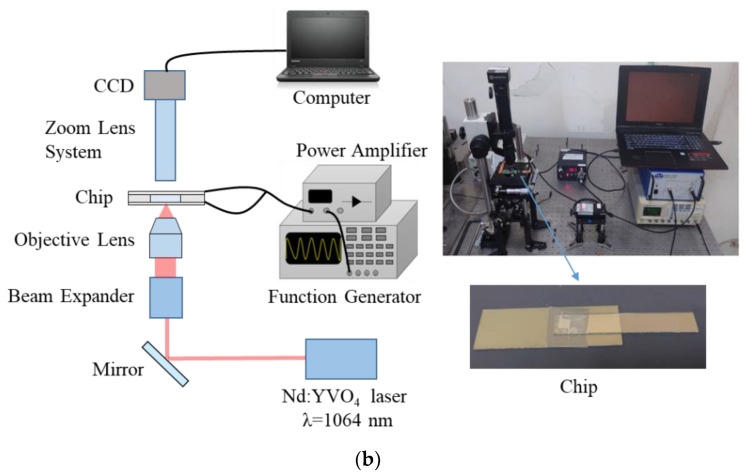
(**a**) Schematic illustration of REP chip. (**b**) Experimental setup and picture of optoelectrokinetic platform.

The chip used for REP manipulation of the polystyrene particles comprised two parallel glass plate electrodes separated by a 100 μm-thick double-sided tape spacer to form a 5 mm × 5 mm (length × width) microchannel chamber. The electrodes were coated with a thin indium tin oxide (ITO) conductive layer with a thickness of 0.7 mm and resistance of 7 Ω (Ritek, Taiwan). Hence, 3 μL of mixed sample consisting of polystyrene beads and DI water was loaded into the microchannel chamber by capillary forces and sandwiched between the two parallel electrode plates. As shown in Figure 1a, the colloidal suspension was subjected to an AC electric field applied between the electrodes and IR laser illumination was simultaneously applied to the lower electrode to generate a local heating effect.

### 3.2. Experimental Setup

Figure 1b shows the self-established experimental arrangement used to manipulate the particles in the microchannel and to observe their motions. The chip was placed on a two-axis stage for particles manipulation. The driving voltage was generated from an AC signal source using a function generator (GFG-3015, GW Instek, Taipei, Taiwan) and was amplified by a power amplifier (MODEL 2100HF, Trek, Lockport, NY, USA) before being supplied to the electrodes of the chip. In addition, a 300 mW power-adjustable Nd-YVO_4_ laser with a center wavelength of 1064 nm (MIL-III-1064, CNI, Changchun, China) was focused on the lower side of the chip. The laser beam was passed through a beam expander (56-30-2-8X, Special Optics, Denville, New Jersey, USA) to produce a parallel beam with a diameter on the cm-scale on the back aperture of the microscope objective lens (UPLFLN 20X, Olympus, Tokyo, Japan). Laser irradiation creates a local temperature gradient in the microchannel chamber coupled with an applied electric field to generate AC electrothermal toroidal vortex drive the particles toward the center of the irradiation region. Furthermore, a zoom lens system was used to obtain a suitable zoom range and resolution of the optical image (6.5X UltraZoom, Navitar, Rochester, NY, USA). The particles were observed using a charge coupled device (CCD) camera (SME-C050-U, Mightex, CA, USA) with a frame rate of up to 14 frames per second (fps) at 2560 × 1920 pixels.

### 3.3. Numerical Simulations

COMSOL Multiphysics (Version 5.3, Burlington, MA, USA) simulations based on the AC/DC, Microfluidics, and Heat Transfer modules were performed to investigate the optoelectrokinetic phenomena (i.e., temperature gradient, electroosmosis, dielectrophoresis, and electrothermal vortex) induced by the REP effect and to explore their effects on the motion and velocity of the particles. The simulations considered the entire geometry of the REP chip and hence a 3D model was used. The colloidal fluid was assumed to be Newtonian and the Brownian motion of the particles was ignored in order to simplify the simulation process. The physical and electrical properties of the conductive fluid (DI water) and particles (polystyrene beads) were listed as Table 1 [21,36,37]. Note that all the internal boundaries were modelled with a no-slip condition. All the external boundaries except the top and the bottom ITO glass plates were modelled with a constant temperature (28 °C) condition. Furthermore, the laser power intensity was set as 75 W/cm^2^ and the amplitude and driving frequency of the AC electric field were set as 35 V_PP_ and 25 kHz, respectively.

## 4. Results and Discussion

### 4.1. Temperature Gradient

**Figure 2:**
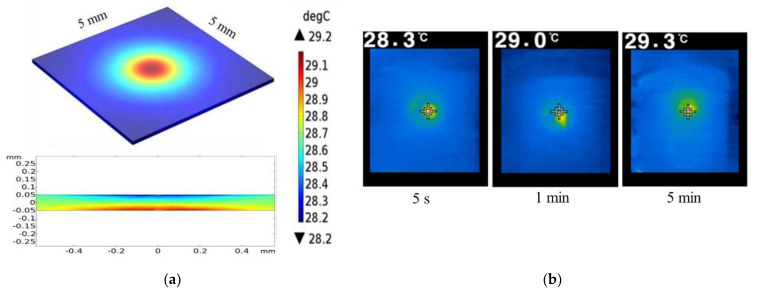
(**a**) Numerical contours of temperature field distribution in liquid layer. (**b**) Experimental temperature measurements in area of laser illumination after 5 s, 1 min, and 5 min. Note that the laser power is 75 W/cm^2^, the voltage amplitude is 35 V_pp_, and the driving frequency is 25 kHz.

The temperature gradient in the chip was generated by a combination of the focused IR laser illumination and the AC electric field. Of the two heating effects, the main contribution to the temperature gradient was provided by the laser illumination, which potentially heats both the substrate and the liquid. Previous studies have shown that the conductivity and permittivity of conductive fluids are both temperature-dependent [26]. Accordingly, the temperature distribution within the chip was simulated using thermal conductivities of 0.35 and 65 W/mK for the DI water and ITO layers, respectively. Figure 2a shows the simulation results obtained for the temperature field distribution in the liquid layer of the chip given the considered laser power of 75 W/cm^2^, driving voltage of 35 V_pp_, and driving frequency of 25 kHz. As shown, the temperature of the liquid layer adjacent to the laser spot is around 29.2 °C and decreases slowly toward room temperature (28 °C) at the upper surface. Figure 2b shows the experimental temperature measurements obtained using a thermal imager (TG165, FLIR Systems, Goleta, CA, USA) after 5 s–5 min of heating. After 5 s, the temperature in the region around the laser illumination spot is approximately 28.3 °C. After 1 min, the temperature in the illumination region reaches 29 °C. However, after 5 min, the temperature saturates at an approximately constant value of 29.3 °C. The experimental temperature measurements are in good agreement with the numerical data, and hence the basic validity of the numerical model is confirmed.

### 4.2. Optoelectrokinetic Synergy Force

**Figure 3:**
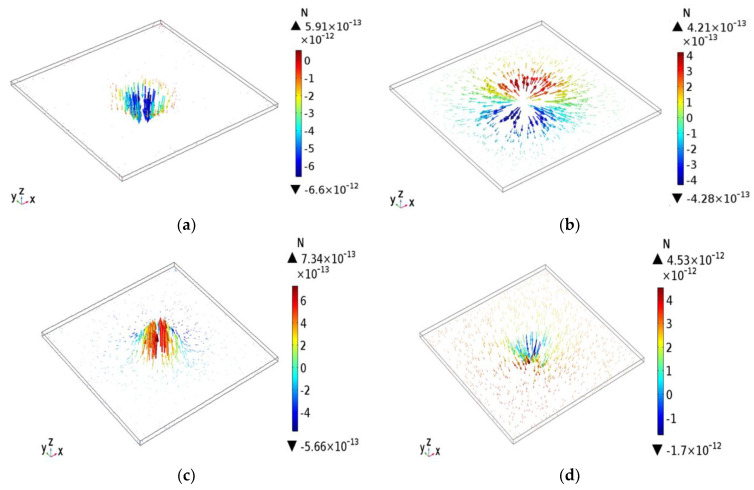
Numerical results for distributions of: (**a**) DEP force, (**b**) thermophoresis force, (**c**) drag force, and (**d**) trapping force acting on 11-μm particles. Note that the laser power is 75 W/cm^2^, the voltage amplitude is 35 V_pp_, and the driving frequency is 25 kHz.

Further simulations were performed to investigate the effects of the synergy force produced by the REP toroidal vortex on the motion of the particles within the microchannel. Figure 3a presents the simulation results obtained for the distribution of the DEP force acting on the polystyrene particles with a diameter of 11 μm. It is seen that the beads experience a negative DEP effect since the dielectric permittivity of the medium (DI water) is larger than that of the particles. In other words, the results confirm that a DEP force is induced, which drives the particles toward the laser illumination spot on the lower side of the chip. For the REP conditions considered in the present case (i.e., a laser power intensity of 75 W/cm^2^ and an amplitude and driving frequency of the AC electric field of 35 V_PP_ and 25 kHz, respectively), the DEP force has a maximum magnitude of 6.6 × 10^−12^ N.

Figure 3b presents the simulation results for the distribution of the thermophoresis force acting on the polystyrene beads. Note that the red arrows represent the thermophoresis force components acting along the coordinate system direction (i.e., x, y, or z axis direction), while the blue arrows represent the thermophoresis force components acting in inverse the coordinate system direction (i.e., -x, -y, or -z axis direction). Note also that the thermophoresis force is governed only by the laser-generated temperature gradient (i.e., it is independent of the applied AC electrical field). The thermophoresis force has a maximum value of 4.28 × 10^−13^ N in the hot-spot region of the microchannel and decreases in the direction of the cooler region of the channel. In other words, the distribution of the thermophoresis force is consistent with that of the temperature field shown in Figure 2a.

From a global perspective (Figure 1a), the AC electrothermal and AC electroosmotic effects drive the fluid toward the center of the irradiation region, resulting in the formation of a re-circulating toroidal vortex flow. Consequently, the particles in the bulk fluid are also transported toward the irradiation region as a result of the hydrodynamic drag force. Figure 3c presents the simulation results for the distribution of the fluid drag force acting on the 11-μm particles. The simulation results confirm that the particles are brought toward the center of the laser irradiation region and then move in the upward direction under the effects of AC electroosmotic. The maximum value of the drag force is 7.34 × 10^−13^ N and occurs near the center of the hot-spot. The drag force acting on the particles comprises three components, namely the AC electrothermal force, the AC electroosmotic force, and the gravity force. When the laser irradiation and electric field are applied simultaneously, the particles can be trapped on the electrode surface within the irradiated spot region under the synergistic action of the light and electric fields [24] (see Figure 1a). Figure 3d presents the simulation results for the distribution of the trapping force acting on the 11-μm particles. Note that the trapping force constitutes the sum of all the forces acting on the particles. It is seen that the particles are brought toward the laser illumination spot on the bottom side of the chip under the effects of the trapping force, and the force has a maximum value of 4.53 × 10^−12^ N in the center region of the hot-spot. Overall, the results confirm the trapping ability of the particles by the optoelectrokinetic effect.

**Figure 4:**
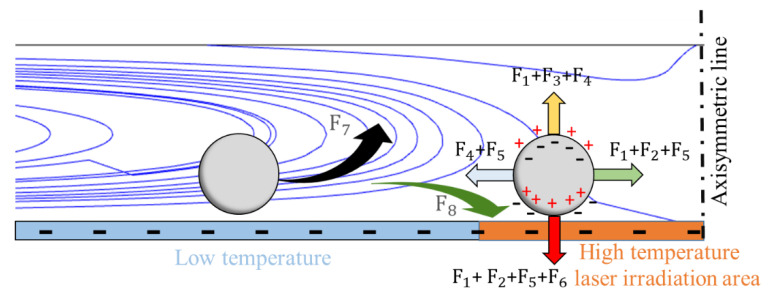
Numerical empirical model used to elucidate relationship among different forces acting in REP chip.

Table 2 lists all the forces acting on the particles in the REP chip. The transport of the particles by the toroidal vortex or trapping of the particles on the heated electrode surface reflects the outcome of a dynamic competition process between the multiple forces [25]. Figure 4 presents a numerical empirical model elucidating the relationships among the forces (Note that the notations refer to the forces listed in Table 2.) As shown, the lift force responsible for moving the particles in the vertical direction away from the bottom electrode comprises the electrothermal force (F_1_), the electroosmosis force (F_3_), and the thermophoretic force (F_4_). The electrothermal flow is the major contributor to the lift force and declines exponentially with an increasing frequency of the AC driving force [16]. Hence, at a certain electrical frequency, the forces acting on the particles balance one another, and the particles aggregate into clusters. Depending on the dielectric properties, shape, and size of the particles, there exists a certain critical electric field under which the particles cannot be trapped in the REP chip [23,24] or circulated continuously within the toroidal vortex structure due to the stronger drag force. However, according to the numerical results presented in Figure 3, for a laser power intensity of 75 W/cm^2^, an electric field strength of 35 V_pp_, and a driving frequency of 25 kHz, the trapping force exceeds the drag force, and hence the 11-μm particles accumulate on the illuminated electrode surface.

### 4.3. Influence Range of Toroidal Vortex

**Figure 5:**
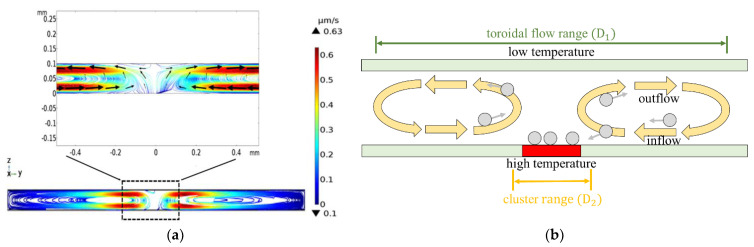

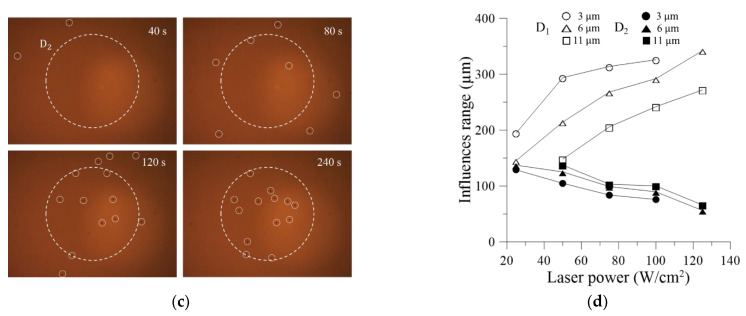
(**a**) Numerical contours and (**b**) schematic illustration showing flow range and clustering range of toroidal vortex structures induced by REP effect. (**c**) Experimental results for trapping of polystyrene beads with diameter of 3 μm at 40, 80, 120 and 240 s, respectively. (**d**) Variation of flow range and clustering range with laser power as function of particle size.

Figure 5a presents the simulations results for the toroidal flow structure induced within the microchannel under a laser power intensity of 75 W/cm^2^, electric field strength of 35 V_pp_, and driving frequency of 25 kHz, respectively. The toroidal flow structure has the form of two symmetrical rotating structures, which drag the particles toward the heated illumination zone of the electrode and then transport them away again after they are pushed away from the lower electrode by the uplift force. The vortex structures rotate in opposite directions and have a maximum hydrodynamic flow velocity of 0.63 μm/s in both cases. The toroidal vortex is induced by several factors, including the dielectric constant, conductivity, and density changes caused by the non-uniform temperature gradient, and acts in competition with the trapping force, which prompts the particles to settle in the hot-spot region of the lower electrode surface. Figure 5b illustrates this competition effect schematically. For certain REP conditions (i.e., laser power intensity, driving voltage and driving frequency), the particles remain in suspension in the bulk fluid and are transported by the rotating vortex structures. The physical range over which the particles are inducted into (and transported by) the toroidal vortexes is defined for convenience as the toroidal flow range (D_1_). However, under certain critical REP conditions, the particles are ejected from the rotating vortex structures and aggregate on the hot-spot region of the lower electrode. The corresponding range over which the clustering effect is induced is referred to as the cluster range (D_2_).

Figure 5c presents the experimental results obtained for the trapping of polystyrene beads with a diameter of 3 μm (shown within the white circles) after REP times of 40, 80, 120 and 240 s, respectively (Note that the laser power intensity is 75 W/cm^2^, the electric field strength is 20 V_pp_, and the driving frequency is 25 kHz.) As shown, the particles barely move before 40 s since the REP effect is not yet fully formed. However, as the REP actuation time increases, the particles within the toroidal flow range are gradually trapped within the cluster range (indicated by the large dashed white circle in each figure) and stop moving completely after around 240 s.

Figure 5d shows the variation of the toroidal flow range (D_1_) and cluster range (D_2_) as a function of the laser power intensity (25~125 W/cm^2^) and particle size (3~11 μm). Note that the electric field has a constant frequency of 25 kHz and a strength of 20, 25, and 35 V_pp_ for particles with a diameter of 3, 6, and 11 μm, respectively. Note also that, to properly explore the competition effect between the toroidal flow process and the clustering process, an adjustment of the electric field strength with the particle size must be performed since higher voltages or laser powers enhance the drag force effect (i.e., the particles keep circulating in the vortex), and hence inhibit the trapping of small particles. By contrast, lower voltages or laser powers inhibit trapping of the large particles due to the lower trapping force. Note that all data are averaged from 10 experiments.

For the smallest particle size of 3 μm, the toroidal flow range (D_1_) has a value of 195 μm for a laser power of 25 W/cm^2^, while the cluster range (D_2_) is equal to 129 μm. When the laser power is increased to 100 W/cm^2^, D_1_ increases to 326 μm and D_2_ deceases to 76 μm. For laser powers greater than 100 W/cm^2^, the particles cannot be trapped and continue circulating within the vortex. For the particles with the largest diameter of 11 μm, the trapping effect is not induced for laser powers lower than 50 W/cm^2^. However, for the maximum laser power intensity of 125 W/cm^2^, D_1_ and D_2_ have values of 272 μm and 66 μm, respectively. In general, the results show that, for a given particle diameter, the toroidal flow range (D_1_) increases with an increasing laser power, while the cluster range (D_2_) decreases. Moreover, the change in the toroidal flow range and cluster range is particularly pronounced for particles with a smaller size. The increase in D_1_ with increasing laser power is the result primarily of the greater temperature gradient induced by a higher laser power, which enhances the size and intensity of the rotating vortex structures.

### 4.4. Particle Velocity

**Figure 6:**
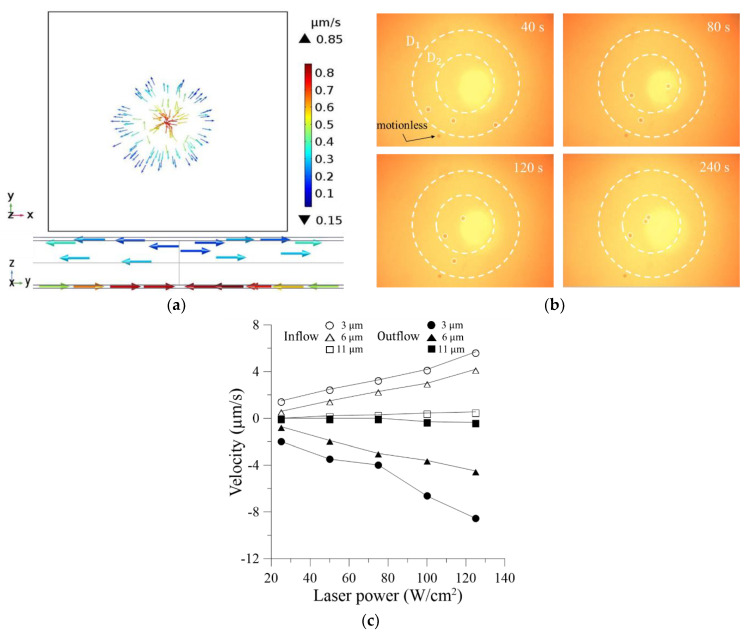
(**a**) Numerical results for velocity field. (**b**) Experimental results for trapping of polystyrene beads with diameter of 11 μm at 40, 80, 120 and 240 s, respectively. Note that the laser power is 75 W/cm^2^, the voltage amplitude is 35 V_pp_, and the driving frequency is 25 kHz. (**c**) Variation of particle velocity with laser power as function of particle size.

Figure 6a presents the simulation results for the distribution of the velocity field acting on the polystyrene beads with a diameter of 11 μm under a laser power of 75 W/cm^2^, electric field strength of 35 V_pp_, and driving frequency of 25 kHz. Note that the color bar and arrows indicate the velocity strength and flow direction, respectively. The maximum particle velocity reaches 0.85 μm/s and the average velocity is around 0.4 μm/s. Furthermore, from the sectional view, it is seen that the velocity of the particles in the lower layer of the channel, which move toward the center of the laser illumination zone, is greater than that of the particles in the upper layer of the channel, which flow away from the center of the laser illumination zone. Figure 6b presents the experimental results obtained under the same REP conditions after 40, 80, 120, and 240 s, respectively. Note that the dashed circles denote the toroidal flow range (D_1_) and cluster range (D_2_), respectively. Note also that the CCD camera is focused on the lower layer of the channel in order to capture the movement of the particles toward the hot-spot region of the lower electrode. Before 40 s, the REP effect is not yet fully formed, and hence the particles remain virtually motionless. However, as the REP time increases, the three particles within the toroidal flow range are transported into the cluster range and stop moving completely after around 240 s. The average inflow velocity of the particles is determined to be around 0.3 μm/s and is thus consistent with the simulation results in Figure 6a.

Figure 6c shows the experimental results for the variation of the mean particle velocity with the laser power (25~125 W/cm^2^) as a function of the particle diameter (3~11 μm). As for Figure 5d, the electric field has a constant frequency of 25 kHz, and the field strength is set as 20, 25, and 35 V_pp_ for the particles with diameters of 3, 6, and 11 μm, respectively. Note that the inflow direction (in the lower layer of the channel) is defined as the positive direction, while the outflow direction (in the upper layer of the channel) is defined as the negative direction. For the particle with a diameter of 11 μm, the average inflow velocity is 0.45 μm/s and the average outflow velocity is 0.3 μm/s for a laser power of 100 W/cm^2^. When the laser power is increased to 125 W/cm^2^, the average inflow velocity and outflow velocity increase slightly to 0.6 μm/s and 0.4 μm/s, respectively. The experimental results are thus in good agreement with the numerical simulation results shown in Figure 6a. For the particles with the smallest diameter of 3 μm, the average inflow velocity is around 1.5 μm/s for the lowest laser power of 25 W/cm^2^, while the average outflow velocity is equal to 2.0 μm/s. For the maximum power of 125 W/cm^2^, the average inflow velocity increases to 5.7 μm/s and the average outflow velocity increases to 8.6 μm/s. Interestingly, the results indicate that the outflow velocity is greater than the inflow velocity for small-sized particles. This finding is reasonable since the DEP force scales proportionally with the third power of the particle radius. Overall, the results indicate that the particle velocity increases with increasing laser power due to the corresponding increase in the intensity of the recirculating vortex structures. The effect of the laser power in increasing the particle velocity is particularly apparent for the small-size particles.

### 4.5. Effect of Driving Frequency on Particle Behavior

**Figure 7:**
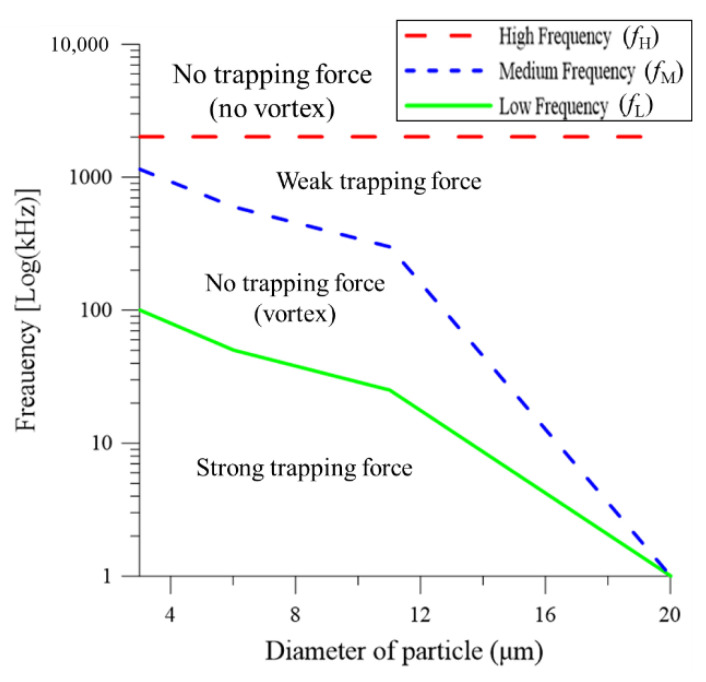
Optoelectrokinetic trapping behavior of particles as function of driving frequency and particle size.

Figure 7 shows the measured optoelectrokinetic behavior of the particles for driving frequencies in the range of 2 kHz~5 MHz. Note that the laser power is 75 W/cm^2^ in every case, and the electric field strength is set as 20, 25, and 35 V_pp_ for the particles with diameters of 3, 6, and 11 μm, respectively (Note that the electric field strength is also set as 35 V_pp_ for the particle with a diameter of 20 μm). All of the particles exhibit a distinct response in different frequency domains, irrespective of their size. Four discrete trapping behaviors are observed, namely a strong trapping force, no trapping force (with vortexes), a weak trapping force, and no trapping force (without vortexes). A preliminary investigation showed that for driving frequencies lower than 900 Hz, bubbles were formed in the colloidal solution, signifying the occurrence of electrolysis due to Faradaic reactions. Accordingly, the minimal frequency for the experimental investigation was set as 2 kHz.

For the smallest particles with a diameter of 3 μm, driving frequencies in the range of 2~100 kHz (*f*_L_, green solid line) cause the particles to aggregate on the irradiated spot and form a dense cluster, as shown in Figure 5c. For driving frequencies of 100~1150 kHz (*f*_M_, blue dotted line), the particle trapping effect temporarily disappears due to the diminished electrical double layer polarization intensity and dielectrophoresis relaxation frequency, and hence the particles continue to be transported in the recirculating toroidal electrothermal flow. In the frequency range of 1150 kHz to 2 MHz (*f*_H_, red dotted line), the particles slowly re-aggregate and form a loose cluster due to the lower intensity of the electrothermal flow as a result of the slow response. The re-aggregation behavior is likely a result of the reduced hydrodynamic lift due to the declining electrothermal flow. A test has been performed by switching off either the laser or the electric fields resulted in disaggregation, confirming the necessity of applying the laser and electric fields simultaneously. The test implied that the particles trapping remained effective but progressively weakened as the frequency approached the upper limit (i.e., *f*_H_), as reported also in [18]. However, for frequencies greater than 2 MHz (>*f*_H_, 2 MHz), the electrothermal flow effect disappears and toroidal vortexes are no longer formed. Hence, no particles are trapped on the irradiated hot-spot region of the electrode. As the particle size increases, the values of *f*_L_ (green line) and *f*_M_ (blue line) reduce, particularly for particle diameters greater than 11 μm. For the particles with the largest size of 20 μm, a trapping effect is not produced irrespective of the value assigned to the driving frequency since the motion of the particles is dominated by the gravity force, and hence the particles simply settle on the electrode surface wherever they happen to be located in the bulk fluid. Overall, the results suggest that the optimal driving frequency for the optoelectrokinetic trapping of the present particles lies within the range of 2~100 kHz. Moreover, the optimal frequency reduces with an increasing particle size.

### 4.6. Particle Sorting

**Figure 8:**
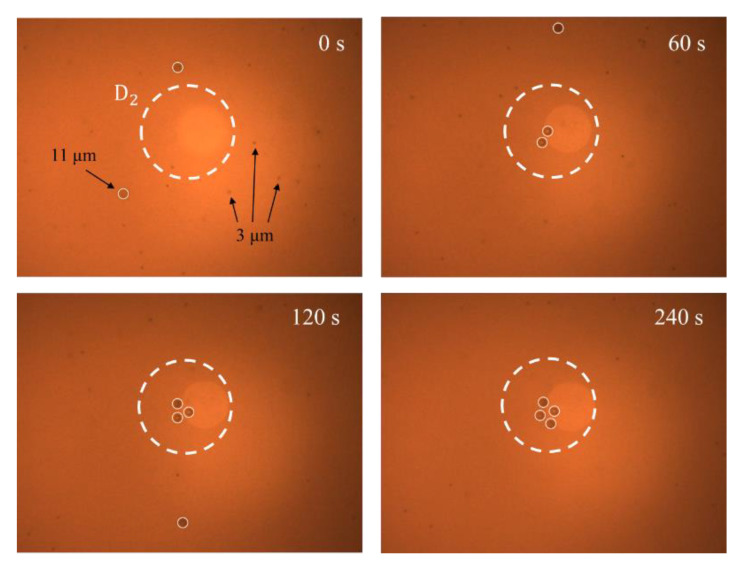
Particle sorting based on voltage modulation effect at 0, 60, 120 and 240 s, respectively. Note that the laser power is 75 W/cm^2^, the voltage amplitude is 35 V_pp_, and the driving frequency is 25 kHz.

A final experiment was performed to evaluate the feasibility for utilizing the REP effect induced under different driving voltages to perform the size-dependent sorting of the particles. A colloidal solution consisting of DI water and a mixture of 3-μm and 11-μm particles was prepared and injected into the microchannel by capillary forces. The laser power and driving frequency were set as 75 W/cm^2^ and 25 kHz, respectively, and the driving voltage was set as 20 V_pp_. The particles with a smaller diameter of 3 μm gradually aggregated within the cluster range (as shown in Figure 5). However, the particles with a diameter of 11 μm remained motionless. When the driving voltage was increased to 35 V_pp_, the 11-μm particles gradually accumulated within the cluster range, while the 3-μm particles circulated continuously within the toroidal vortex structure due to the stronger drag force. The corresponding results are shown in Figure 8, in which the 11-μm particles are circled for ease of visualization, while the 3-μm particles are shown in black and are distributed over the toroidal flow range (Note that the focus plane of the CCD camera was adjusted to capture the motion of the heavier 11 μm particles, and hence the 3-μm particles appear slightly blurry in the images). The experimental images presented in Figure 8 confirm the feasibility for sorting the particles based on their size.

## 5. Conclusions

This study has conducted a numerical and experimental investigation into the manipulation of polystyrene beads within a REP chip consisting of a 100-μm deep microchannel sandwiched between two ITO-coated parallel plate glass electrodes. The simulation results have shown that under appropriate REP conditions (i.e., certain values of the laser power intensity, AC electric field voltage, and AC electric field frequency), recirculating toroidal vortex structures are induced, which transport the particles within the bulk fluid such that they first approach and then move away from the illuminated hot-spot region on the lower electrode. However, at certain critical values of the REP parameters, a trapping force is also induced, which causes some of the particles to be ejected from the recirculating vortex structures and to accumulate on (or close to) the hot-spot region. The outcome of the competition process between the drag force induced by the rotating vortex structures and the trapping force induced by the particle-electrode interactions in the vicinity of the hot-spot is dominated by the effect of the DEP force, which scales proportionally with the third power of the particle radius. The simulation results showed that the trapping force (4.53 × 10^−12^ N) acted on the 1-μm particles more than the drag force (7.34 × 10^−13^ N). Therefore, the particles are brought toward the laser illumination spot on the bottom side of the chip given the considered laser power of 75 W/cm^2^, driving voltage of 35 V_pp_, and driving frequency of 25 kHz. The experimental results show that the range over which the toroidal vortex transports the particles increases with an increasing laser power intensity and reducing particle size. By contrast, the range over which the particles are trapped and cluster on the hot-spot region of the lower electrode reduces as the laser power increases due to the corresponding increase in the intensity of the recirculating flow structures. The cluster range similarly reduces with a reducing particle size since the particles with a smaller dimension (and hence weight) are more readily transported by the toroidal vortex structures.

The experimental results have shown that the particle velocity increases with an increasing laser power and a reducing particle size. Moreover, for a constant laser power, the velocity of the particles toward the center of the laser illumination zone in the lower region of the microchannel is greater than that of the particles moving away from the laser illumination zone in the upper layer of the channel for larger particles. However, an opposite tendency is observed for the particles with a smaller dimension. For a constant laser power and driving voltage, the particles experience four different trapping effects as the driving frequency increases, namely (1) a strong trapping force, (2) no trapping (with vortexes), (3) a weak trapping force, and (4) no trapping (without vortexes). Generally speaking, the threshold frequencies associated with the different trapping responses reduce with an increasing particle size. For the particles considered in the present study with a diameter in the range of 3~11 μm, the optimal frequency range was shown to be 2~100 kHz. Finally, the experimental results demonstrate the feasibility of performing a size-dependent sorting of the particles by setting an appropriate value of the REP driving voltage (i.e., 35 V_pp_ for the present colloidal solution containing a mixture of 3-μm and 11-μm particles).

## Figures and Tables

**Table 1:** A list of parameters used in REP modeling.

	ρ(kg/m^3^）	ε	σ(S/m)	c(J/kgK)	*κ*(W/mK)
Fluid	1000	78	$5.5\times 10^{-6}$	4200	0.6
Particle	1040	2.7	0.0009	1300	0.033

**Table 2:** Summary of forces acting on particles in REP chip.

Force	Description
F_1_	AC Electrothermal, ACET
F_2_	Dielectrophoresis, DEP
F_3_	AC Electroosmosis, ACEO
F_4_	Thermophoretic force
F_5_	Electrical double layer, EDL
F_6_	Gravity
F_7_	Drag force (F_1_+F_3_+F_6_)
F_8_	Trapping force (the sum of all forces)

## Data Availability

Not applicable.

## References

[1] Suscillon C., Velev O.D., Slaveykova V.I. (2013). Alternating current-dielectrophoresis driven on-chip collection and chaining of green microalgae in freshwaters. Biomicrofluidics.

[2] Kim H.T., Bae H., Zhang Z., Kusimo A., Yu M. (2014). Optofluidic microvalve-on-a-chip with a surface plasmonenhanced fiber optic microheater. Biomicrofluidics.

[3] Huang H.Y., Huang Y.H., Kao W.L., Yao D.J. (2015). Embryo formation from low sperm concentration by using dielectrophoretic force. Biomicrofluidics.

[4] Li S., Li M., Bougot-Robin K., Cao W., Chau I.Y.Y., Li W., Wen W. (2013). High-throughput particle manipulation by hydrodynamic, electrokinetic, and dielectrophoretic effects in an integrated microfluidic chip. Biomicrofluidics.

[5] Williams S.J., Kumar A., Wereley S.T. (2008). Electrokinetic patterning of colloidal particles with optical landscapes. Lab Chip.

[6] Demircan Y., Ozgur E., Kulah H. (2013). Dielectrophoresis: Applications and future outlook in point of care. Electrophoresis.

[7] Juan M.L., Righini M., Quidant R. (2011). Plasmon nano-optical tweezers. Nat. Photonics.

[8] Ashkin A., Dziedzic J.M., Yamane T. (1987). Optical trapping and manipulation of single cells using infrared laser beam. Nature.

[9] Zheng T., Wang C., Xu C., Hu Q., Wei S. (2018). Patterning microparticles into a two-dimensional pattern using one column standing surface acoustic waves. Sens. Actuators A Phys..

[10] Ebrahimian H., Giesguth M., Dietz K.-J., Reiss G., Herth S. (2014). Magnetic tweezers for manipulation of magnetic particles in single cells. Appl. Phys. Lett..

[11] Cummins Z., Probst R., Shapiro B. (2013). Electrokinetic tweezing: Three-dimensional manipulation of microparticles by real-time imaging and flow control. Lab Chip.

[12] Velasco V., Williams S.J. (2013). Electrokinetic concentration, patterning, and sorting of colloids with thin film heaters. J. Colloid Interface Sci..

[13] Ndukaife J.C., Mishra A., Guler U., Nnanna A.G.A., Wereley S.T., Boltasseva A. (2014). Photothermal heating enabled by plasmonic nanostructures for electrokinetic manipulation and sorting of particles. ACS Nano.

[14] Zhang H., Liu K.-K. (2008). Optical tweezers for single cells. J. R. Soc. Interface.

[15] Chiou P.Y., Ohta A.T., Wu M.C. (2005). Massively parallel manipulation of single cells and microparticles using optical images. Nature.

[16] Kumar A., Williams S.J., Wereley S.T. (2009). Experiments on opto-electrically generated microfluidic vortices. Microfluid. Nanofluid..

[17] Williams S.J., Kumar A., Green N.G., Wereley S.T. (2010). Optically induced electrokinetic concentration and sorting of colloids. J. Micromech. Microeng..

[18] Wang K.C., Kumar A., Williams S.J., Green N.G., Kim K.C., Chuang H.S. (2014). An optoelectrokinetic technique for programmable particle manipulation and bead-based biosignal enhancement. Lab Chip.

[19] Liang W., Liu L., Wang J., Yang X., Wang Y., Li W.J., Yang W. (2020). A review on optoelectrokinetics-based manipulation and fabrication of micro/nanomaterials. Micromachines.

[20] Chen W.L., Jayan M., Kwon J.S., Chuang H.S. (2021). Facile open-well immunofluorescence enhancement with coplanar-electrodes-enabled optoelectrokinetics and magnetic particles. Biosens. Bioelectron..

[21] Kumar A., Cierpka C., Williams S.J., Kähler C.J., Wereley S.T. (2011). 3D3C velocimetry measurements of an electrothermal microvortex using wavefront deformation PTV and a single camera. Microfluid. Nanofluid..

[22] Kwon J.S., Wereley S.T. (2015). Light-actuated electrothermal microfluidic motion: Experimental investigation and physical interpretation. Microfluid. Nanofluid..

[23] Williams S.J., Kumar A., Green N.G., Wereley S.T. (2009). A simple, optically induced electrokinetic method to concentrate and pattern nanoparticles. Nanoscale.

[24] Kumar A., Kwon J.S., Williams S.J., Green N.G., Yip N.K., Wereley S.T. (2010). Optically modulated electrokinetic manipulation and concentration of colloidal particles near an electrode surface. Langmuir.

[25] Kwon J.S., Wereley S.T. (2013). Towards new methodologies for manipulation of colloidal particles in a miniaturized fluidic device: Optoelectrokinetic manipulation technique. J. Fluids Eng..

[26] Mishra A., Kwon J.S., Thakur R., Wereley S. (2014). Optoelectrical microfluidics as a promising tool in biology. Trends Biotechnol..

[27] Mishra A., Gupta K., Wereley S.T. (2021). Nature of trapping forces in optically induced electrothermal vortex based tweezers. Phys. Rev. Fluids.

[28] Kunti G., Agarwal T., Bhattacharya A., Maiti T.K., Chakraborty S. (2020). On-chip concentration and patterning of biological cells using interplay of electrical and thermal fields. Anal. Chem..

[29] Gupta K., Chen Z., Williams S.J., Wereley S.T. (2021). Time-resolved particle image velocimetry analysis and computational modeling of transient optically induced electrothermal micro vortex. Electrophoresis.

[30] Kunti G., Bhattacharya A., Chakraborty S. (2018). Electrothermally actuated moving contact line dynamics over chemically patterned surfaces with resistive heaters. Phys. Fluids.

[31] Kunti G., Mondal P.K., Bhattacharya A., Chakraborty S. (2018). Electrothermally modulated contact line dynamics of a binary fluid in a patterned fluidic environment. Phys. Fluids.

[32] Ramos A., Morgan H., Green N.G., Castellanos A. (1999). AC electric-field-induced fluid flow in microelectrodes. J. Colloid. Interface. Sci..

[33] Hughes M.P., Pethig R., Wang X.B. (1996). Dielectrophoretic forces on particle in traveling electric fields. J. Phys. D Appl. Phys..

[34] Jerome B., Mykolas Z., Robin L., Yang L., Erika E. (2017). Colloidal motion under the action of a thermophoretic force. J. Chem. Phys..

[35] Liu S., Lin L., Sun H.B. (2021). Opto-thermophoretic manipulation. ACS Nano.

[36] Kim D., Shim J., Chuang H.S., Kim K.C. (2015). Numerical simulation on the opto-electro-kinetic patterning for rapid concentration of particles in a microchannel. Biomicrofluidics.

[37] Chiou C.H., Chien L.J., Lin J.L., Kuo J.N. (2016). Novel electrodeless dielectrophoresis device for nanoparticle trapping using three dimensional inverted-pyramid arrays. Appl. Phys. Express.

